# Periodontal Wound Healing and Tissue Regeneration: A Narrative Review

**DOI:** 10.3390/ph14050456

**Published:** 2021-05-12

**Authors:** Young-Dan Cho, Kyoung-Hwa Kim, Yong-Moo Lee, Young Ku, Yang-Jo Seol

**Affiliations:** Department of Periodontology, School of Dentistry and Dental Research Institute, Seoul National University and Seoul National University Dental Hospital, Seoul 03080, Korea; cacodm1@snu.ac.kr (Y.-D.C.); perilab@snu.ac.kr (K.-H.K.); ymlee@snu.ac.kr (Y.-M.L.); guy@snu.ac.kr (Y.K.)

**Keywords:** periodontium, periodontal tissue regeneration, wound healing

## Abstract

Periodontal disease is a major public health issue, and various periodontal therapies have been performed to regenerate periodontal tissues. The periodontium is a complex structure composed of specialized tissues that support the teeth, and most periodontal surgeries are invasive procedures, including a resection of the gingiva or the alveolar bone. The periodontal wound healing process is slightly different from cutaneous wound healing and is similar to fetal healing, being almost scar-free. The aim of this review article is to provide an overview of periodontal wound healing and discuss various surgical and pharmaceutical approaches to achieve stable wound healing and improve the treatment outcomes. In addition, detrimental and limiting factors that induce a compromised prognosis are discussed, along with the perspective and future direction for successful periodontal tissue regeneration.

## 1. Introduction

**Figure 1:**
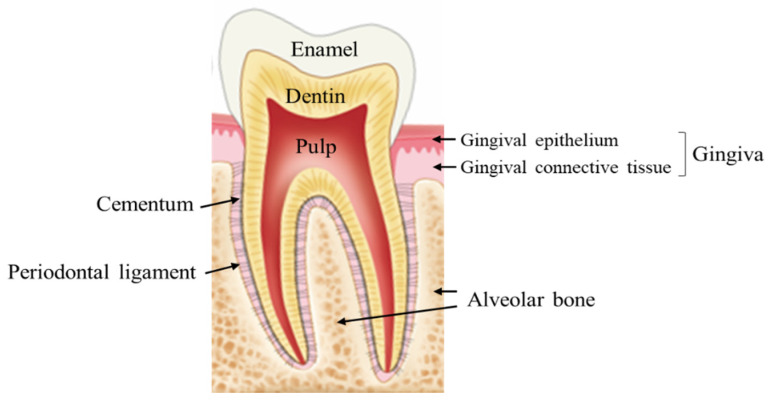
The structure of periodontium.

The periodontium acts as a supporting apparatus for the teeth and is a complex structure consisting of soft and hard tissues [1]. The main functions of the periodontium are to ensure that the teeth are attached to the bones; to protect the nerves, blood vessels, and teeth from injuries; and to provide a barrier to the underlying structures from the oral microbiome. Soft tissue includes the gingiva, mucosa, and periodontal ligament (PDL), and hard tissue includes the cementum and the alveolar bone (Figure 1).

The gingiva is a covering tissue that protects the alveolar bone from external stimuli such as microbial attack or mechanical force and consists of epithelial and connective tissues. The general risk factor of periodontal disease is oral microbial infection, which can induce inflammation of the gingiva, and if not treated on time, it affects the periodontium in general [2,3]. Periodontal disease is the most common oral disease with a high prevalence of 20–50% of the world population [4]. Gingivitis and periodontitis are typical periodontal diseases [1]. Gingivitis is the mildest form of periodontal disease, which is limited to gingival inflammation and is reversible [5]. Conversely, periodontitis is irreversible, accompanied by alveolar bone defect, can induce tooth mobility when untreated, and finally, leads to tooth extraction [6]. Periodontal defect is caused by chronic pathological conditions, such as periodontitis, and exhibits the loss of gingiva, periodontal ligament, and alveolar bone. Various procedures to support the regeneration of the periodontal tissue as well as therapeutics to enhance the wound healing process have been studied. A specialized oral environment with saliva and oral microorganisms can affect the defect formation and wound healing process. The objective of this review is to provide an overview of periodontal wound healing and discuss the complications and factors influencing the healing process and therapeutics for improving periodontal wound healing.

## 2. Normal Wound Healing

**Figure 2:**
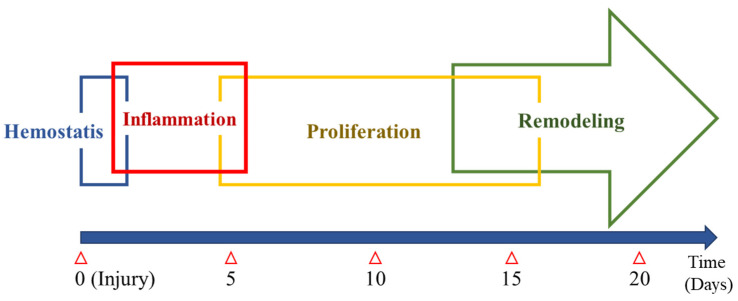
Wound healing process. After injury, wound healing begins with the following process: 1. hemostasis and coagulation, 2. inflammation, 3. cell proliferation, and 4. wound remodeling and maturation.

Wound healing is a dynamic process, which has been a challenge in the clinical setting after treatment. Much effort has been focused on wound management for developing healing techniques and new treatment approaches. Basically, the wound healing process consists of four distinct but overlapping phases [7]: 1. hemostasis and coagulation, 2. inflammation, 3. cell proliferation, and 4. wound remodeling and maturation (Figure 2). This general principle of wound healing also applies to periodontal wound healing [8]. The wound healing procedure involves several types of cells, extracellular matrix, cytokines, and growth factors. Understanding wound healing with regard to various aspects of cells, molecules, physiology, and biochemistry is important to regenerate tissues that are functionally and structurally indistinguishable from the original tissue and not repaired tissue with fibrotic scars. If there is an injury that damages the vessel, hemorrhage is the first process to begin on the wound site. Under normal conditions, a molecular machinery for blood clot formation is immediately operated, protecting the injury site and serving the provisional matrix for cell migration. Blood clot formation continues to the initial inflammatory stage, in which inflammatory cells, including polymorphonuclear neutrophils and monocytes, are activated. These cells clean the wounds of necrotic tissue and bacteria and secrete various enzymes for wound debridement. This inflammatory response shifts into the late phase where macrophages move into the wound area and secrete cytokines or growth factors for the cells involved in the wound healing process. Following the inflammatory stage, granulation tissue formation is initiated with collagen accumulation. Cytokines and growth factors by macrophages induce the migration and proliferation of fibroblasts and endothelial cells into the wound site. This cell-rich granulation tissue activates the phase of matrix formation and maturation. Fibroblasts replace the provisional extracellular matrix by producing a new collagen-rich matrix, and the endothelial cells are involved in angiogenesis for vascularization. Subsequently, wound epithelization is conducted by epithelial cells from the basal layer. The granulation tissue maturation leads to tissue regeneration or repair, which is decided by two main factors: available cells and cell recruiting signals. The skin and gingiva, typical body covering tissues, are considered structurally and functionally homogenous tissues showing similar healing patterns in response to injury. Both are characterized by the presence of keratinized epithelium with underlying connective tissue, which acts as a barrier to microorganisms and other contaminants [9].

## 3. Distinct Characteristics of the Oral Wound

The cutaneous and gingival tissues follow similar macroscopic healing patterns—hemostasis, inflammation, proliferation, and remodeling of the collagen—but are accompanied by microscopic changes at the molecular level and show a distinguished healing response. In the oral cavity, wound healing occurs in warm mouth fluids containing millions of oral microorganisms that might be perceived as detrimental to the healing process [10]. Nevertheless, wounds in the oral cavity heal much faster compared to skin wounds, with rapid re-epithelialization and re-modeling resulting in minimal scar formation [11]. This advantage could be explained by the presence of growth factors or cytokines in the saliva and the important role of fibroblasts. Saliva contains a number of important molecules, such as epidermal growth factors, lysosomes, and lactoferrin, which have antimicrobial and anti-inflammatory properties [12]. Fibroblasts are the main cells of gingival tissue and play an important role in wound healing, especially extracellular matrix (ECM) remodeling by the synthesis of ECM components (such as collagen, fibronectin, hyaluronan, and elastin) and the secretion of matrix metalloproteinase and tissue inhibitor of metalloproteinase [13]. The only exception in the healing potential is excisional wounds on the hard palate. The connective tissue is very thin at palate; therefore, the depth of the wound can reach to the surface of cranial bone and the healing is slow [14]. Recent research has revealed the molecular differences between oral and skin wound healing. Most studies have suggested that an oral wound is in the “primed” state for wound healing compared to a skin wound (Table 1) [9,15,16,17].

### 3.1. Attenuated Inflammatory Reaction

Regarding the phenomenon of scarless and rapid healing, some studies have tried to explain it using the similarity of oral and fetal wounds that includes decreased levels of pro-inflammatory cytokines, such as IL-6 and IL-8 [11], and inflammatory cells, including macrophages and neutrophils [18], but the healing process is not identical. Compared to adult skin wounds, fetal wounds showed decreased levels of transforming growth factor-β1 and increased IL-10; however, no change was seen in oral wounds. Additionally, other studies have suggested positive effects on the inflammation of saliva containing growth factors, protease inhibitors, and cytokines [19]; however, a clear explanation is still not characterized with controversial results [20]. Further in-depth research about the critical difference of oral wounds could provide good candidate biomarkers for wound healing acceleration materials.

### 3.2. Differential Angiogenesis Pattern

Compared to skin wounds, oral wounds exhibit reduced levels of vascular endothelial growth factor expression and more muted angiogenic response, which was supported by dissimilar levels of hypoxia and hypoxia inducible factor-1alpha (HIF-1α) expression. Skin wounds have been shown to be significantly more hypoxic and to have higher levels of HIF-1α than oral wounds [21]. However, this finding of decreased angiogenesis could be open to controversy, since unwounded oral mucosa is more vascularized than the skin [18].

## 4. Periodontal Treatment and Wound Healing

In periodontal surgery, the wound healing pattern is largely divided into two types: primary and secondary healing [22]. Primary healing is a proper procedure for wound healing, which is performed when the gingival tissue is perfectly replaced or closely approximated in the same position pre- and post-operatively. The healing pattern shows rapid healing time with little or no scarring and low infection risk. Conversely, secondary healing occurs when the wound site is not covered by epithelial tissue due to accidents (rupture of suturing and loss of covering materials, etc.) or intention (apically positioned flap and tooth extraction socket, etc.).

### 4.1. Tooth Extraction

**Figure 3:**
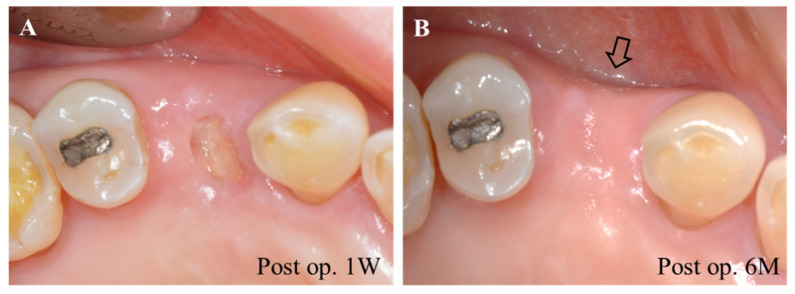
Extraction socket healing. (**A**) Re-epithelization is in progress at post op. 1 week. (**B**) Alveolar bone loss is observed after 6 months of socket healing; the convex alveolar bone has become concave (arrow).

Tooth extraction is one of the most common surgical procedures performed in dental clinics. When diagnosed with hopeless tooth condition due to periodontitis, dental caries, trauma, etc., tooth extraction is performed. The subsequent process of socket healing has become an important subject of research and clinical practice for the successful healing and restoration in the missing region with dental implants. The socket healing pattern post tooth extraction follows a bone healing process with a series of orderly biological events. In general, after that tooth is isolated from the socket in the alveolar bone, the socket is filled with blood clots. Re-epithelization is progressed around 24 h after extraction, and then blood clots are replaced by granulation tissue. The extraction socket is gradually filled with bone, and the bone remodeling process proceeds around 6 months after extraction (Figure 3). The process of socket healing has been widely studied in human and animal models, such as canines, rats, and mice, with radiographical and histological methods. Although the sequence of biological events was similar, a difference was seen in healing time [23]. The socket healing process is accompanied by the loss of alveolar bone height and width due to bone remodeling, including bone formation and resorption. The degree of bone resorption during the remodeling procedure depends on various factors, including local factors such as the quality and quantity of alveolar bone, the presence of inflammation, the use of grafting materials, and oral hygiene, and systemic factors such as smoking, nutrition, and medical condition. A fresh extraction socket, such as extraction due to dental caries or tooth fracture, mostly follows a favorable healing procedure; however, a periodontally compromised socket with severe bone defect by chronic pathologic lesion can result in erratic healing by connective tissue infiltration instead of bone formation [24].

### 4.2. Resective Periodontal Surgery

In the treatment of patients with periodontitis, surgical intervention (such as scaling and root planing) could be required after completion of the non-surgical phase. A periodontal flap is a section of gingiva or mucosa which is surgically separated from the underlying tissues such as the alveolar bone and connective tissue. The periodontal flap operation enables access to root surfaces for cleaning and to remove inflamed soft or hard tissue to reestablish a stable periodontal structure. Depending on the bone exposure after flap reflection, the periodontal flaps are classified as partial-thickness or full-thickness flaps. For favorable healing, the entire surgical procedure should be planned in detail with regard to type of incision, flap design, flap closure, suture, etc., before the procedure. The flap design with a sharp and correct incision is important to preserve good blood supply for the maintenance of vascularization and a reduction in postoperative shrinkage, and an exact flap position with stable suturing is crucial for the stability of the blood clot in the wound site. To improve the healing potential, minimally invasive surgery (MIS), a surgical technique to minimize flap reflection, has been introduced to reduce the wound size and healing time with decreased pain and infection risk. MIS is defined as “refinements in existing basic surgical techniques that are made possible by the use of surgical microscopes and subsequent improved visual acuity,” and three principles are required to be fulfilled [25]: 1. enhancing the surgical ability by the improvement of motor skills, 2. exact primary apposition of the wound edge by passive wound closure, and 3. the application of micro surgical instruments and sutures to reduce tissue trauma. Based on this concept, in 2007, Cortellini and Tonetti suggested the minimally invasive surgical technique (MIST) in periodontal surgery [26], and the use of a microscope and microsurgical instrument was necessary to increase the surgical prognosis. Several studies have shown the advantages of MIST, which include rapid wound healing with less granulation or scar tissue formation and less inflammation or pain.

### 4.3. Regenerative Periodontal Surgery

**Figure 4:**
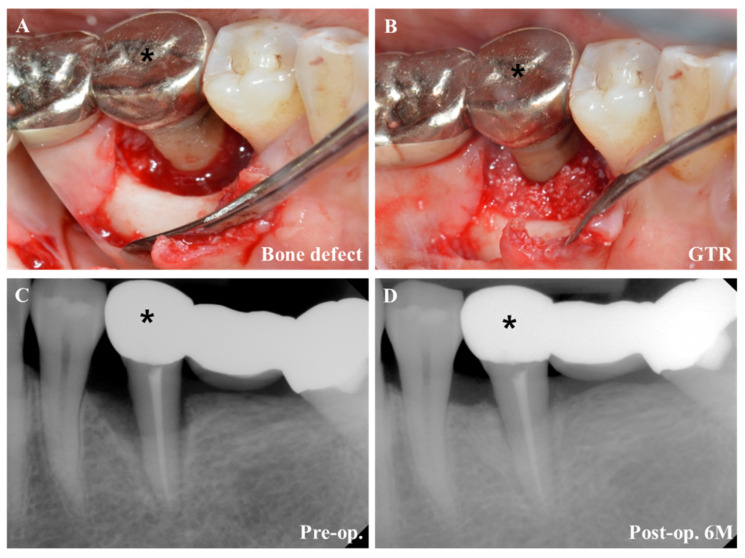
GTR. (**A**) Severe alveolar bone defect around tooth (*) is observed. (**B**) After cleaning the defect, bone grafting was performed. (**C**) Preoperative condition observed in radiograph. (**D**) Postoperative periodontal tissue regeneration was observed in radiograph.

The treatment of periodontal disease has been gradually shifting from resective to regenerative therapy with progress in the understanding of periodontal wound healing. The development of biomaterials, instruments, and techniques has supported the paradigm shift focusing on actual periodontal tissue regeneration, including functional PDL formation [27]. Periodontal tissue regeneration is largely classified into guided tissue regeneration (GTR) and guided bone regeneration (GBR). Simply, GTR refers to the regeneration of periodontal attachment including bone, PDL, and cementum, whereas GBR refers to works on the edentulous area, such as bone grafting and ridge augmentation. GTR is a representative procedure of regenerative surgery in the periodontally compromised site and is defined as follows: “procedures attempting to regenerate lost periodontal structures through differential tissue response” [28] (Figure 4). In periodontitis, alveolar bone resorption is accompanied with gingival inflammation, which causes the breakdown of periodontal attachment. Theoretically, periodontal defects (including soft tissue and hard tissue) cannot be regenerated to the original structure by repair. For successful regeneration, cells that have the ability to form cementum, PDL, and bone should move to the defect site and activate the differentiation potential from progenitor cells. However, practically, epithelial cells move rapidly to the defect site and inhibit the proliferation and differentiation of progenitor cells for the maturation of cementum, PDL, and bone; eventually, the repair would be superior to the regeneration. Therefore, the concept of “guide” using a barrier was introduced for selective cell repopulation, proliferation, and differentiation. As a barrier, resorbable or non-resorbable membranes have been widely used to prevent gingival epithelium or connective tissue entering the bony defect site and to induce osteogenesis and PDL regeneration. The membrane creates a space acting as a scaffold for vascularization and cell ingrowth from the base of the periodontal defect, prevents bacterial invasion, and isolates the inner space from undesirable cells after therapy. Furthermore, it provides a good environment for favorable wound healing, acting as a double layered gingival flap to improve the stability of the blood clot and protect the interface between the root surface and healing tissues, preventing the rupture of the surgical wound site.

## 5. Complications after Periodontal Treatment

A complication is a secondary disorder arising as a consequence of the primary disease or condition. The complications arising after periodontal therapy include postoperative infection, bleeding, swelling, pain, bruising, and adverse tissue changes. Among them, the first three can interfere with the wound healing procedure and alter the treatment outcomes [29].

### 5.1. Postoperative Infection

Due to the distinct character of the oral cavity—an open space toward the outside—and food intake, infection could be a risk factor inhibiting the normal wound healing process. Oral microbiome on the wound bed is one of the most critical barriers for the infection. The wound healing process could be impaired by pathological microorganisms that produce free-radicals, destructive enzymes, and toxins and down-regulate the immune response and inhibit collagen formation. Generally, the occurrence of bacteremia depends on the degree of trauma during treatment and could be prevented by prophylactic antibiotics. Amoxicillin is widely used as the first choice for infection prophylaxis [22].

### 5.2. Bleeding

Postoperative bleeding is always followed at varying degrees. Within 12 h of surgery, some bleeding is considered normal; however, persistent hemorrhage or oozing could be problematic to wound healing [30]. Postoperative bleeding is classified into primary, reactionary, and secondary hemorrhage. Primary hemorrhage occurs at the time of operation, reactionary hemorrhage occurs 2–3 h after operation due to the loss of vasoconstrictor effect under anesthesia, and secondary hemorrhage occurs up to 2 weeks after the operation due to infection. In uncontrolled hemorrhage, various methods are applied for bleeding control. The hemostatic agents typically used are silver nitrate and ferric sulphate, and materials include oxidized cellulose, suturing, and collagen or gelatin sponges. Furthermore, bone wax is used to prevent bleeding from bones, and electric devices used to seal the damaged vessel are effective in the control of extensive bleeding. Hemostasis in patients with a bleeding disorder or taking anticoagulants should be handled with great caution. Preoperative cessation of anticoagulants and the preparation of blood transfusion in case of emergency are considered.

### 5.3. Swelling

Swelling is defined as the enlargement of a body part as a result of inflammation or filling with tissue fluids and is considered to be a normal postoperative reaction until it interferes with wound healing [31]. This expected swelling is generally related to surgical range and operating time, and especially associated with increased blood supply to the surgical site. If swelling persists, the wound site can reopen despite the suture, and primary healing will not be possible. The administration of antibiotics and steroids is recommended pre- or post-operatively to prevent unexpected swelling from disturbing the process of healing [32].

### 5.4. Scar Formation

Scar formation, known as fibrosis, is an inevitable result of cutaneous wound healing; however, it varies widely depending on the anatomical sites. Scar formation occurs when normal physiological process is not achieved. Most oral wounds do not cause serious scars; however, fibrosis can occur when healthy bone does not support the base of the wound, such as in congenital defects, lip clefts, and/or palate clefts [33]. Additionally, a persistent inflammation in the chronic wound can result in delayed healing and fibrosis [16].

## 6. Factors Affecting Periodontal Wound Healing

It is important to recognize risk factors related to the wound healing process to achieve favorable wound healing after treatment.

### 6.1. Vascularization, Flap Design, and Incision

In periodontal surgery, a comprehensive anatomical understanding is significant for flap design with incision. To prevent complications in wound healing and angiogenesis, an accurate cognition on the branching of periosteal vessels is required [34,35]. It is widely accepted that flap elevations without a vertical incision benefit from accelerating blood supply and favorable wound healing, resulting in the improvement of esthetic outcomes, better patient satisfaction, and minimal risk of scarring [34,36]. In particular, scarring on the anterior part by impaired blood supply creates an esthetic problem. Vertical incision, such as a coronally advanced flap in vertical bone augmentation, is unavoidable, and the incision should be placed at the mesial side of the flap to avoid the interruption of vascular flow from the posterior end to the anterior end of the scar [37]. Additionally, long time periodontal surgery with a vertical incision and additional local anesthetics such as epinephrine can induce ischemia in the mucogingival flap [38,39].

### 6.2. Aging (Senescence)

Aging is a biological process characterized by a decrease in cellular function that is induced by a gradual deficiency in regenerative reactions with a dramatic change in gene expressions in body tissues [40]. After tissue injury, various types of cells (neutrophils, lymphocytes, monocytes, fibroblasts, endothelial cells, and keratinocytes) are recruited, which secrete and organize the components of the ECM, including collagen, proteoglycan, and fibronectin. Normal periodontal wound healing requires normal reactive cells and a healthy ECM; however, aging affects both cell response and physiology of the ECM unfavorably [41]. Differences in the wound healing process by aging are as follows. In the inflammatory phase of wound healing, aging may prolong the production of inflammatory cytokines, which delay wound healing and tissue fibrosis, reducing the regenerative potential. Senescence-associated secretory phenotype (SASP) is characterized by a proinflammatory trait of senescent cells, with the involvement of CCAAT/enhancer and nuclear factor-kB (NF-kB) associated with the secretion of chemokines, cytokines, and proteolytic enzymes. Following the altered inflammatory phase, new tissue formation and remodeling phases are also affected. Aging decreases collagen synthesis, cell migration, proliferation, and differentiation in the new tissue formation phase and increases the production and activity of matrix metalloproteinases (MMP) and apoptosis in the tissue remodeling phase [42].

### 6.3. Diabetes Mellitus (DM)

DM is a representative disease exhibiting an impairment in wound healing [43]. This impaired healing process in DM patients involves complex pathophysiological mechanisms. Hypoxia, which is induced by insufficient perfusion or angiogenesis, is a critical risk factor in DM that amplifies early inflammatory responses, thus increasing the production of oxygen radicals [44]. Hyperglycemic conditions aggravate wound healing with hypoxia by increasing the oxidative stress and formation of advanced glycation end-products [45]. High levels of MMP and the dysfunction of fibroblasts and keratinocytes inhibit the normal repair process, resulting in tissue destruction. Dysregulated immune-related cell functions, including defects in bactericidal capacity, leukocyte chemotaxis, phagocytosis, and dysfunctions of fibroblasts, also add to impaired repair. Additionally, neuropathy in DM may contribute to impaired wound healing with a decrease in neuropeptides, including substance P, calcitonin related peptide, and nerve growth factor [46].

### 6.4. Smoking

The negative effects of smoking on wound healing are well known [47]. In periodontal surgeries, including dental implant placement, impaired healing in smokers has been reported [48,49,50,51]. Nicotine, a major component of tobacco, is quickly absorbed by diffusion through the buccal mucosa and produces a variety of systemic effects [52,53]. Reportedly, nicotine inhibited the fibroblast activities of fibronectin and collagen synthesis, and increased collagenase activity, inducing collagen degradation [52,54]. In periodontal disease, gingival bleeding is lesser in smokers than in non-smokers because of decreased gingival blood flow due to nicotine’s vasoconstrictive effect. This effect leads to insufficient vascularization of the gingiva, in turn lowering the wound healing potential and increasing the chance of bacterial infection [55]. Additionally, Imamura et al. reported that nicotine reduced epithelial cell migration, which is important for re-epithelialization during wound healing, through MAPL ERK1/2 and p38 signaling pathways [56]. Based on this evidence, smoking delays wound healing and adds various complications, such as wound rupture, infection, tissue necrosis, and epidermolysis [57].

## 7. Therapeutics for Periodontal Wound Healing

### 7.1. Biopharmaceutical Approaches

#### 7.1.1. Enamel Matrix Derivative (EMD)

EMD was introduced as a tissue healing agent derived from proteins during cementogenesis in the tooth development to stimulate tissue regeneration. Amelogenin is the main protein component of EMD, and EMD has been focused in GTR in particular because it promotes PDL fibroblast proliferation and inhibits epithelial growth, which is a key and necessary mechanism in GTR. Emdogain^®^ (Straumann, Switzerland) is a commercial product containing a mixture of EMDs that promote periodontal tissue regeneration in the application at the root surface and have osteopromotive properties [58,59]. Furthermore, Emdogain^®^ enhances wound healing in the gingival tissue with reduced complications, such as inflammatory reaction and pain, even in skin wound healing [60,61]. In vitro and in vivo studies have shown that EMD reduces the secretion of chemokines and pro-inflammatory cytokines related to chemotaxis, angiogenesis, inflammation, and fibroplasia, and its effect is critical in early wound healing according to a clinical study [61]. Diabetes is one of the main risk factors for wound healing, and Takeda et al. reported that EMD promotes periodontal tissue regeneration through the Akt/VEGF signaling pathway even in diabetic patients [62].

#### 7.1.2. Collagen

Collagen is a naturally available, as well as the most abundant, protein present in the extracellular matrix and acts as an important component of physical and functional structure in the body [63]. With its chemotactic character, collagen improves the fibroblasts’ migration, proliferation, and differentiation of specialized cells. Additionally, collagen plays an important role in wound healing, including platelet activation and angiogenesis [64]. In addition to these advantages, collagen has been widely used in various formulations in periodontal surgery due to its easy manipulation. However, its hydrophilic properties can act as a disadvantage, leading to rapid enzymatic degradation and swelling. Collagen membrane is used as a barrier to prevent the preoccupancy of epithelial cells in the periodontal defect site in GTR (Table 2). Various types of membranes used in GTR derived from different animals (porcine, bovine, and equine) consist of type I or III collagen or both. Collagen is also applied to the tooth extraction socket for bleeding control and blood clot stabilization. This hemostatic collagen is highly absorbent; therefore, it absorbs the blood as soon as it is applied to the bleeding site, serving as a mechanical obstruction. Furthermore, when collagen comes in contact with blood, it causes platelet aggregation, releasing thromboxane A2, and creates an artificial clot-like structure.

#### 7.1.3. Blood-Derived Products

Blood-derived products, including platelet rich plasma (PRP), plasma rich growth factor (PRGF), and fibrin sealant, have been used in regenerative surgical procedures. The preparation method is based on concentrating platelets, leukocytes, and growth factors, but their contents are slightly different. PRP consists of platelets and leukocytes, but PRGF does not have leukocytes. Fibrin sealant is derived from blood plasma, which is cryoprecipitated to obtain fibrinogen.

1. PRP

PRP is an autologous bioactive substance that has various applications in the medical and dental field, including plastic surgery and periodontal or maxillofacial surgery for the enhancement of wound healing. PRP is obtained from the middle layer of white blood cells and platelets in centrifugated blood. Platelets contain biologically active proteins that bind to the fibrin mesh or ECM and recruit stem cells that promote wound healing [86]. In periodontal treatment, PRP is easily applied to soft or hard tissue therapy. In hard tissue application, PRP enhances the healing of intrabony defects when combined with bone grafting [87]. In soft tissue application, several clinical reports suggested the advantages of PRP, such as accelerated wound healing and improved esthetics [88]. However, the efficacy of PRP in periodontal treatment has been controversial due to diverse or adverse clinical outcomes. PRP may not provide additional effects compared to GBR in relation to dental implants, and the evidence of effects of PRP in sinus elevation seem weak [89]. This may be due to different platelet numbers or PRP concentration related to growth factors [90]. PRP has been somewhat advantageous when used for periodontal tissue regeneration; however, enough controlled clinical trials have not been conducted to prove its efficacy.

2. PRGF

PRGF is a second-generation blood-related product, similar to PRP, and requires less venous blood, making it convenient, time-consuming, safe, easy-to-use, and fast healing [91]. In contrast to PRP, PRGF does not contain white blood cells and associated inflammatory byproducts for avoiding the proinflammatory effects of proteases and acid hydrolases [92]. Plasma-derived adhesive molecules, such as fibronectin, fibrinogen, and vitronectin, serve as a matrix for attracting progenitor cells, and platelet concentrates act as reservoirs for growth factors, such as platelet derived growth factor (PDGF), insulin-like growth factor (IGF), transforming growth factor (TGF), and vascular endothelial growth factor (VEGF). PRGF induces fibroblast proliferation and epithelial tissue healing. Due to its gel-like consistency, it seems more ideal for non-surgical application than surgical. In randomized clinical trials, the clinical efficacy of PRGF as an adjunct to non-surgical periodontal treatment has been proved with a reduction in pocket depth and gain in clinical attachment level [91]. Even in surgical procedures, the use of liquid PRGF accelerates bone healing in the extraction socket and sinus lift and promotes the osseointegration of dental implants [88].

3. Fibrin Sealant

The proteins fibrin and fibronectin play a crucial role in hemostasis and wound healing. If an injury occurs, fibrinogen is activated by thrombin to form insoluble fibrin clots for hemostasis. Wound healing is processed via interactions of cell surface receptors with fibrin and other proteins, including fibronectin. Fibronectin is an adhesive glycoprotein with multiple binding sites for surface receptors on various cells, such as fibroblasts [93]. Fibrin sealant, known as glue, has been used in surgeries for hemostasis and anchoring the graft. The application of fibrin sealant in periodontal surgery also showed excellent hemostatic and tissue adhesive effects [94]. The product of fibrin sealant is composed of thrombin and fibrinogen. When they are mixed together, fibrin clot formation is conducted by the induction of the final stage of the blood clotting pathway. Fibrin sealant enhances mechanical strength and blood clot stability and can be prepared from autologous blood without an immune reaction.

### 7.2. Periodontal Dressing Materials

**Figure 5:**
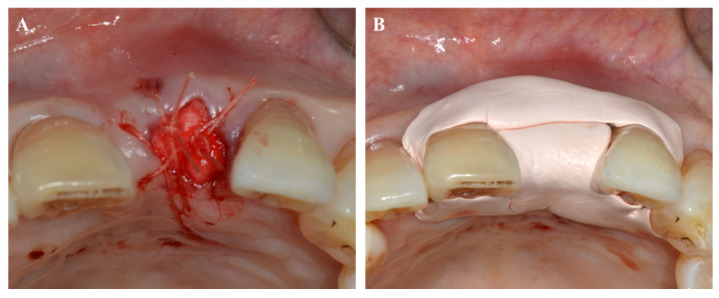
Periodontal dressing application. (**A**) After tooth extraction, collagen material was applied to the extraction socket. (**B**) Coe-Pak was applied to the surgical site to protect the wound.

Periodontal dressing has been introduced for the protection of wounds by preventing post-operative hemorrhage, irritation, and microbial contamination in the surgical site. The first use of a periodontal dressing with iodoform gauze was reported, and then the first commercial product, “Wonderpak,” which consists of zinc oxide, eugenol, pine oil, alcohol, and asbestos fibers, was introduced. The two types of periodontal dressings are largely classified according to the gradients: 1. zinc oxide eugenol (hard type) and 2. zinc oxide non-eugenol (soft type). Zinc oxide non-eugenol dressings are more commonly used because zinc oxide eugenol has disadvantages such as hardness, difficulty of adaptation, burning sensation, or allergic reaction from unreacted eugenol. A widely used non-eugenol dressing is Coe-Pak (Dentsply, Germany), which consists of two types of pastes—base and accelerator (Figure 5). In addition, dressings without zinc oxide or eugenol, such as light curing or collagen-containing periodontal dressings, are also available. Due to its transparent property, a light curing dressing is applied to the anterior part. Collagen-containing dressing promotes wound healing by deposition of the fibers in the granulation tissue. In the past, despite the advantages of periodontal dressing, whether to use or not use it was debatable. The type of dressing used for a wound seems subjective. Presently, it might be accepted that if adequate primary closure of the gingival flap is achieved, there is little necessity to use dressing, but the protection of the surgical area with dressing is recommended when secondary healing is anticipated.

### 7.3. Devices to Improve Wound Healing

#### 7.3.1. Light Amplification by Stimulated Emission of Radiation (Laser)

The laser has three key elements: power (thermal property), wavelength (optical property), and pulse or wave (operating mode). Two types of lasers are available depending on tissue penetration depth. High-intensity laser therapy (HILT), such as neodymium-doped yttrium-aluminum-garnet (Nd:YAG), carbon dioxide (CO_2_), erbium, and diodes, is used in the surgical process of not only ablation, vaporization, and coagulation but also the stimulation of wound healing. Conversely, low-level lasers and light-emitting diodes (LEDs) are used as “biostimulators” with lower power than that of surgical lasers. Photobiomodulation therapy (PBMT) is defined as light therapy using low-level lasers and LEDs that promote wound healing by inducing epithelial cell proliferation, anti-inflammatory response, pain relief, and inhibition of scar formation [95]. In dentistry, lasers have been widely used in periodontal treatment (Table 3). The best advantage of HILT is hemostasis with easy ablation of the soft tissue. Compared to blade incision, HILT can easily cut and reshape the gingival tissue with reduced pain, bleeding, and suturing [96]. In addition, PBMT is a unique aspect of lasers, which promotes wound healing and reduces inflammation and pain [97,98]. The U.S. Food and Drug Administration (FDA) defined the therapeutic lasers with wavelengths of less than 500 mW as harmless, classified them as “low-risk devices,” and proposed the guideline for laser application in dentistry; the coagulation of extraction sites using diode lasers and CO_2_ lasers. The advantage of therapeutic lasers is that they stimulate natural biological processes and mainly affect cells with oxidation–reduction (redox) reactions. Healthy cells do not respond strongly to lasers because they cannot increase their redox ability immediately, while damaged cells with low redox level will be quickly stimulated. Light photons absorbed by the mitochondria of the cells increase the amount of adenosine triphosphate, a cellular energy, via the removal of nitric oxide on cytochrome c oxidase and generate reactive oxygen species, which eventually stimulates cell signaling and gene expression-related wound healing.

#### 7.3.2. Hyperbaric Oxygen

Hypoxia is a microenvironmental feature of inflammatory disease, wound healing, and cancers, in which the demand of O_2_ is higher [105]. Hypoxia and low O_2_ pressure have a negative effect on the function of inflammatory cells and fibroblasts during the healing procedure [106]. Hyperbaric oxygen therapy (HBOT) is used for therapeutic purposes with 100% O_2_ under a certain pressure. HBOT increases the oxygen tension in the arterial blood and sequentially improves the cellular oxygen tension, which stimulates angiogenesis and wound healing, and has bactericidal or bacteriostatic effects [107,108]. For these reasons, the use of HBOT has been applied as an adjunctive therapy in periodontal treatment; however, most are case reports [109,110]. Further controlled studies are needed to confirm the clinical potential of HBOT.

## 8. Perspective and Future Directions

Wound healing in the oral cavity is similar and different in many ways from skin wound healing. Successful periodontal wound healing after treatment can support optimal periodontal tissue regeneration. In-depth understanding of the biological factors affecting periodontal therapy has always been emphasized to optimize the clinical outcome and increase the predictability of therapy. Adjunctive use of biomaterials or devices can reinforce the healing process, and wound biomodification using therapeutic agents, such as growth factors, may amplify the regenerative potential.

Stem-cell related research has received a lot of attention for the bright future of periodontal tissue regeneration; however, several problems remain to be solved. The development of appropriate scaffolds that deliver the cells and growth factors even in the infective healing environment (due to the oral microbiome) is important for genuine tissue engineering. A well-organized strategy for the periodontal healing process is needed since the advantages of high healing potential and disadvantages of oral microbial attacks are combined. With recent ongoing advanced technologies of three-dimensional printing and next-generation sequencing for personalized medicine, ideal wound healing and tissue regeneration would be more achievable.

## Figures and Tables

**Table 1:** Characteristics and differences of oral and skin wounds.

	Oral Wound	Skin Wound
**Re-epithelialization**	Re-epithelialization in oral wound is faster than skin wound	
Re-epithelialization (24 h)	100%	40%
**Inflammation**	Inflammatory reaction is reduced and resolution is faster in oral wound than skin	
Inflammatory cells (Neutrophils, T cells, Macrophages)	↓	↑
Cytokines (IL-1β, IL-6, IL-α, TNF-α)	↓	↑
**Angiogenesis**	Angiogenic response is decreased in oral wound	
Vessel density	↓	↑
VEGF	↓	↑
**ECM**	MMT/TIMP ratio is decreased in oral wound	
Matrix metalloproteinases (MMP)	↓	↑
Tissue inhibitor of metalloproteinase (TIMP)	↑	↑
**SCAR**	Reduced scar formation is observed in oral wound	
TGF-β1/β3	↓	↑

**Table 2:** Clinical application of collagen in periodontal treatment.

Types	Application	Commercial Product (Manufacturer)	Reference
**Sponge**	- Hemostasis - Reduction in pain - Soft tissue contouring - Wound dressing - Socket grafting	CollaPlug (Integra LifeSciences Corp.)	[65,66]
		OraPlug (Salvin)	[67]
		Teruplug (Olympus Terumo Biomaterials)	[68,69,70]
		Avitene Ultrafoam Collagen Sponge (Davol, Inc.)	[71,72]
**Membrane**	- Barrier in GTR or GBR	Bio-Gide (Geistlich)	[73,74,75,76,77,78]
		BioMend/OsseoGuard (Zimmer Biomet Inc.)	[73,78,79]
		Ossix (Datum Dental Ltd.)	[80,81]
		Periogen (Collagen Corporation)	[73,82]
		CollaCote/CollaTape (Integra LifeSciences Corp.)	[83,84,85]

**Table 3:** Dental application of therapeutic laser.

Application	Type	Method	Effect	Ref.
Extraction socket	Combined HILT and PBMT	HILT (27 J) was performed immediately after tooth extraction to enhance blood coagulation, followed by PBMT (0.7 J) 1 day later to enhance healing	Combined HILT and PBMT following tooth extraction hastened wound healing and preserved alveolar crest height, suggesting a role in socket preservation	[99]
Recurrent aphthous stomatitis (RAS)	CO2 laser, Nd:YAG laser and diode laser	Laser treatment included Nd:YAG laser ablation, CO2 laser applied through a transparent gel (non-ablative) and diode laser in a low-level laser treatment (LLLT) mode	The use of lasers (CO2 laser, Nd:YAG laser and diode laser) to relieve symptoms and promote healing of RAS	[100]
Inflammatory fibrous hyperplasia	Diode laser systems	Randomized, split-mouth clinical trial; comparative evaluation of diode laser and scalpel surgery	Bleeding and bacterial count was low in the laser group	[101]
Frenectomy	Nd:YAG laser treatment	Randomized clinical trial on postoperative discomfort after Nd:YAG laser and conventional frenectomy	Nd:YAG laser treatment used for frenectomies provides better postoperative comfort (pain, chewing, talking)	[102]
Harvesting de-epithelialized palatal graft	Diode laser systems	Randomized clinical trial: comparative evaluation of diode laser and scalpel surgery	Laser technique decreased post-operative morbidity	[103]
Free gingival graft	PBMT	A split-mouth triple-blind randomized controlled clinical trial	PBMT accelerated the rate of epithelialization at the donor site	[104]

## Data Availability

Not applicable.
